# Regulating Genome Editing: For an Enlightened Democratic Governance

**DOI:** 10.1017/S0963180118000403

**Published:** 2019-01

**Authors:** GIULIA CAVALIERE, KATRIEN DEVOLDER, ALBERTO GIUBILINI

**Keywords:** genome editing, public engagement, public policy, democracy, moral psychology, moral pluralism

## Abstract

How should we regulate genome editing in the face of persistent substantive disagreement about the moral status of this technology and its applications? In this paper, we aim to contribute to resolving this question. We first present two diametrically opposed possible approaches to the regulation of genome editing. A first approach, which we refer to as “elitist,” is inspired by Joshua Greene’s work in moral psychology. It aims to derive at an abstract theoretical level what preferences people would have if they were committed to implementing public policies regulating genome editing in a context of ethical pluralism. The second approach, which we refer to as the democratic approach, defended by Francoise Baylis and Sheila Jasanoff et al., emphasizes the importance of including the public’s expressed attitudes in the regulation of genome editing. After pointing out a serious shortcoming with each of these approaches, we propose our own favored approach—the “enlightened democracy” approach—which attempts to combine the strengths of the elitist and democratic approaches while avoiding their weaknesses.

With every significant scientific breakthrough that could have significant societal impacts, such as the development of a new biotechnology, ethical questions typically arise at two levels. At the first level, there are *substantive ethical* questions, such as questions regarding the moral status of the new biotechnology and the ethical dilemmas that may arise from its application. Questions at the second level pertain to how we should regulate this biotechnology and who should decide about how to regulate it. We will refer to these as *ethical-political questions*.

Within society and among bioethicists, there is often significant disagreement at the level of substantive ethical questions, which is not surprising given that our views are influenced by highly diverse political, cultural, moral, and religious beliefs. Persistent disagreement at this level is not necessarily problematic. The coexistence of fundamentally different ethical views is not only inherent to democratic societies^1^; it is often considered essential for them to thrive.^2^ Arguably more problematic is disagreement at the level of ethical-political questions, as this could hinder the policy-making process and leave controversial biotechnologies unregulated, or regulated in a problematic way, e.g., in a way that alienates a large section of society.^3^ This is, arguably, what happened with the regulation of genetically modified (GM) foods.^4^ Governments and scientists have been criticized for not taking the public’s concerns about GM foods sufficiently seriously and for misidentifying the nature of their concerns, which then contributed to a lack of public support for the development of this technology, and to a general mistrust in science.^5^

The recent debate on genome editing raises concerns previously discussed in the debates on GM foods and rDNA experimentation,^6^ as well as new questions arising from the efficacy, precision, and relatively low cost of application of genome editing.^7^ The possibility of inserting heritable changes in human embryos has attracted the most attention. Some argue that genome editing in human embryos should be pursued, as it could prevent particular genetic diseases from being passed on from one generation to the next.^8^ Others warn that it will be too difficult to (ever) assess the technology’s safety, and that, therefore, we should probably not pursue it.^9^ In addition, manipulating human genes more generally has been criticized on the ground that it will erode the intrinsic value of what is naturally produced,^10^ will be tantamount to “playing God,”^11^ or will result in the resurgence of eugenics.^12^

The debate on genome editing has so far primarily focused on ethical questions at the substantive ethical level, that is, questions about the moral status of genome editing and, especially, its application in human embryos. Limited attention has been devoted to questions regarding its regulation.^13^ This limited attention is problematic, given the pressing need to regulate genome editing technologies and the potential negative societal impact of regulations that alienate large swaths of society.

Because the debate on genome editing is just emerging, there is an opportunity to approach it with fresh eyes and to shape it in a way that includes discussions of ethical-political questions. In our view, such inclusion would ensure that regulations on genome editing take societal views into account, something which, as we will argue, is of the utmost importance if we want to implement ethically acceptable policies. Discussions of ethical-political questions regarding genome editing can also inform wider debates on the regulation of science in democratic societies characterized by a plurality of coexisting views. As Sheila Jasanoff et al. have pointed out, genome editing raises “basic questions about the rightful place of science in governing the future in democratic societies,”^14^ and as Francoise Baylis has suggested, genome editing offers an opportunity to rethink existing mechanisms of public engagement and to identify the “common interests that might rightfully guide policy deliberations.”^15^

We take on the challenges that Baylis and Jasanoff et al. identify by exploring possible approaches to regulating genome editing that lie at the opposite ends of what we might call “the spectrum of public involvement in policy making.” We first sketch an approach that does not take into account the *actual* preferences of those potentially affected by genome editing and its regulation, but rather aims to derive at an abstract theoretical level what preferences people would have if they were committed to implementing public policies in a context of ethical pluralism; this is a strategy proposed by Joshua Greene. After pointing out a serious shortcoming of this approach, which we dub “elitist,” we present a diametrically opposed approach, as defended by Baylis and Jasanoff et al. We refer to this approach as “democratic,” as it emphasizes the importance of including the public’s expressed attitudes in the regulation of genome editing. We conclude that this approach also has a serious shortcoming and propose our own favored approach, the “enlightened democracy approach,” which attempts to combine the strengths of the elitist and democratic approaches without their weaknesses. Our approach is inspired by the literature on deliberative democracy.^16^ It relies on a democratic process as well as on expertise to identify people’s preferences and to develop policies that reflect them.

## The Elitist Approach

Ideally, *since we live in democratic societies and we value democracy*, genome editing should be regulated in a way that all people *can* agree upon. However, universal or even very widespread agreement is unlikely to occur, given that views at the level of substantive ethical questions tend to influence those at the level of ethical-political questions. Typically, those who think it is morally desirable, or even morally obligatory, to pursue genome editing will favor permissive regulations,^17^ whereas those objecting to applications of the technology, or to the technology itself, will favor more restrictive regulations^18^

How then are we to make progress at the level of ethical-political questions?

One approach we could adopt is to leave aside the expressed views on the regulation of genome editing and determine what people *would* agree upon *under ideal conditions*.^19^ What these ideal conditions are is of course up for debate, but throughout history, many philosophers have focused on the relevance of reason, or rationality, to the resolution of ethical questions.

For example, Baruch Spinoza wrote that “men who are governed by reason—that is, who seek what is useful to them in accordance with reason, desire for themselves nothing, which they do not also desire for the rest of mankind, and, consequently, are just, faithful, and honorable in their conduct.”^20^ The idea is that people “governed by reason” will agree upon universal norms that would apply to themselves as well as to others. Unfortunately, moral philosophers who have tried to ground their proposed ethical theories on the basis of reason alone have failed to reach an agreement on what reason requires or on what the rational—and therefore the ethical—way to regulate human behavior is. Indeed, the two main normative ethical theories that both claim to be grounded in rationality—Kantianism and utilitarianism—are often taken to lie at opposite ends of a spectrum, one grounding a strictly deontological approach and one grounding a strictly consequentialist one. How, then, could we rely on reason or rationality to determine how we should regulate genome editing? We could turn to political philosophers, but it seems like the best we can do then is to agree to disagree and to accept disagreement among reasonable ethical views within a framework of political liberalism.^21^ However, what liberal policies should admit as a reasonable view turns out to be difficult to establish, in particular when the disagreement is so deep that it involves not only substantive ethical views but also ethical-political views. What kind of principles can reasonably settle a disagreement about how to regulate a technology whose moral status is the subject of substantive ethical disagreement? The answer remains unclear.

Perhaps we could turn to moral psychology for help. Recent work in moral psychology, particularly with regard to the interplay between reason and moral intuitions and emotions in our moral and political judgments,^22^ could potentially support ethical theories grounded in reason or rationality. In other words, an understanding of how moral judgments are formed could perhaps inform an account of how rationality could allow us to find some form of agreement at the ethical political level in the face of persisting and unresolvable disagreement at the substantive ethical level. In the remainder of this section, we focus primarily on how the work of Greene in moral psychology could underpin an approach that seeks to determine what regulations on genome editing people would agree upon if they were governed by reason. We do appreciate that Greene’s work is debated on methodological and normative grounds,^23^ and we do not commit ourselves to his particular approach to the formation of moral judgments. What we are offering here is merely one possible heuristic that could underpin the “elitist” approach, and what we say is compatible with rejecting some specifics of Greene’s model.

On the basis of magnetic resonance imaging (MRI) studies and psychological experiments involving people’s responses to variations of the so-called trolley problem,^24^ Greene has developed a dual-process model of how people’s moral judgments are formed.^25^ On Greene’s model, there are two modes of making (moral) judgments: an automatic and a manual mode (what Daniel Kahneman would call “thinking fast” and “thinking slow”). In everyday situations, we normally make moral judgments in automatic mode, that is, on the basis of intuitive and emotive responses (such as the judgment that it is wrong to push a man onto the track so that he would stop a trolley and prevent five people from being killed). Such automatic mode is the result of how morality evolved to facilitate cooperation with other members of the small groups, or “tribes,” within which individuals used to live. Responding to ethical dilemmas in automatic mode has resulted in different “tribes,” or different moral communities, developing different intuitive and emotive responses (e.g., more conservative, or more liberal) to ethical dilemmas.^26^ This automatic mode coexists with the manual mode, which is guided by more reasoned reflections that can obtain once people set aside their intuitive and emotive responses.^27^ According to Greene, the manual mode is what one could and should rely upon when it comes to solving moral conflicts arising between different moral communities. Such conflicts arise frequently today because of the globalized world in which we live, which often requires individuals belonging to different moral communities to find common solutions to ethical problems arising from the application and regulation of new technologies. Genome editing might well be one example.

Greene is convinced that if we could set aside our intuitive and automatic responses to the ethical questions that divide us, and reflect on these questions with the aid of our reflective cognitive capacities (the manual mode), we would be able to formulate a “metamorality,” that is, a “shared moral standard”^28^ that is genuinely based on reason.^29^ The metamorality would be a “global moral philosophy that can adjudicate among competing interests of its members” and that would allow “trade-offs among competing tribal values.”^30^ In order to make these trade-offs, however, we need a common currency of value that all human beings can acknowledge, even if it conflicts with some of the views developed in automatic mode.^31^ Thus, even if some people disagree on the shared moral standard identified (due to their automatic moral mode), everyone should be able *to understand* (due to their manual moral mode)^32^ this standard and its relevance for approaching ethical disputes. So, how to find this shared moral standard?

According to Greene, adopting the manual mode and favoring reasoned reflection instead of automatic intuitive responses to ethical questions allows us to appreciate that there are two essential aspects of a genuinely ethical approach. The first is the value of impartiality—the idea that, from the point of view of the universe (so to speak), each person is as important as any other. Greene acknowledges that none of us are really truly impartial, but notes that we can all acknowledge the importance of impartiality as a moral ideal.^33^ The second aspect of a genuinely ethical approach is the recognition of the value of happiness, which matters to everyone.^34^ Recognizing that happiness is what ultimately matters and that, from the point of view of the universe, no one matters more than anyone else, lies at the core of utilitarianism, which Greene proposes to rename “deep pragmatism.” This is to emphasize that it is the approach that is most likely to work in resolving moral conflicts because it is the one on which people from different moral tribes could get to agree upon once they switch from the automatic to the manual mode of reasoning.

So, how could an approach based on Greene’s ideas about how to resolve moral disagreement in a globalized world help us regulate genome editing? Policies would need to be developed using the utilitarian standard. In other words, alternative regulatory strategies would need to be evaluated on the basis of their capacity to generate the greatest happiness for the greatest number, as the famous utilitarian slogan goes. However, whether different types of policies to regulate genome editing can be expected to maximize happiness is a question that is not easily settled. Different sorts of experts, including for instance legal experts, policy-makers, scientists, ethicists, and sociologists could contribute to the assessment of the expected consequences of potential regulatory strategies, of what “happiness” could mean, and of how the consequences could contribute to the promotion of happiness. (Within this framework, a relevant and philosophically interesting question that would need to be addressed, but which we raise here only to leave aside, is one about the proper role of “moral experts,”^35^ i.e., people who know well different possible moral theories and know how to weigh conflicting moral values against one another in the light of those moral theories.)^36^ Presumably, these experts would be people who are able to switch to the manual mode and set aside automatically formed intuitions and emotions. Because the proposed approach heavily relies on some sorts of experts, we propose to refer to it as an “elitist approach.”

In principle, this approach could be the ethically optimal solution to the moral disagreement about how to regulate genome editing: it would be the solution that perfectly rational people would endorse. However, there are also some serious shortcomings with this view, which make it a problematic approach to regulating genome editing.

## A Shortcoming of the Elitist Approach

We focus our criticism on an elitist approach modeled on Greene’s proposal, but our arguments would also apply to other similarly elitist approaches.^37^

The most serious shortcoming is that the elitist approach is not democratic, in the sense that the decision-making process does not require the involvement and participation of all those who will be affected by the decisions taken. Why is this problematic?

Democratic decision-making procedures can be important for intrinsic reasons, for example because one values equality in political influence and sees democracy as the only system that can respect and preserve people’s freedom, equality, and equality in freedom.

But a democratic decision-making procedure can also be important for instrumental reasons, because it is essential to achieve trust and legitimacy, which both have desirable consequences for society.^38^ Relying on an elitist approach to regulate genome editing excludes large segments of the population from the decision-making process. Expertise can often be “exclusionary and restricted,” as it represents “the command of knowledge within a defined domain by some persons that is not commanded by others.”^39^ As a result, those excluded may lose trust in the policies resulting from the elitist approach and in the various experts that have contributed to them. Loss of trust in experts may have a wide societal impact. Moreover, when legitimacy^40^ obtains, people are more inclined to conform to the policies and to avoid forgoing the potential benefits the technology in question may bring about. As is often highlighted in the literature on trust and expertise, it would be difficult for science to make progress without this trust^41^ and without legitimacy.^42^ In addition, it has been argued that relying on a democratic process is good because involving rival points of view is more likely to lead to better policy outcomes, given that different ethical and practical problems are more likely to be considered and analyzed.^43^

These reasons point to something similar: in liberal, democratic societies, public policies, and political decisions in general, cannot do without some form of support by the people who will be affected by those policies.

## The Democratic Approach

This importance of relying on a democratic process to regulate genome editing echoes a shared view among the few scholars that have addressed the level of ethical-political questions specifically regarding genome editing^44^ (and indeed, some preliminary experiments of public dialogue in this direction have been carried out).^45^ It has been argued that an absolute condition of developing policies to regulate this technology is public engagement and the inclusion of public views in policy-making processes. Institutional bodies such as the U.S. National Academy of Sciences or the U.K. Nuffield Council on Bioethics endorse this view. For instance, following the December 2015 International Summit on Genome Editing, the National Academy of Sciences Organizing Committee released a statement that called for the establishment of an “ongoing international forum to discuss potential clinical uses of gene editing.” According to the statement, this forum should be “inclusive among nations” and should

Engage a wide range of perspectives and expertise—including from biomedical scientists, social scientists, ethicists, health care providers, patients and their families, people with disabilities, policymakers, regulators, research funders, faith leaders, public interest advocates, industry representatives, and members of the general public.^46^

Echoing this conclusion, Baylis emphasizes the need to collectively discuss strategies for governance that are based on a “broad consensus” which, in turn, should be achieved through “broad-based participation by persons from around the world with a range of perspectives and interests.”^47^

An even more radical position is expressed by Jasanoff et al., who openly criticize the reliance on experts to address the regulatory challenges raised by genome editing and argue that public engagement cannot be reduced to asking questions to the public that have been preselected, preapproved and deemed appropriate by experts. They claim that

Even where there are calls for “broad public dialogue,” these are constrained by expert accounts of what is proper (and not proper) to talk about in ensuing deliberations. When larger questions arise, as they often do, dissent is dismissed as evidence that publics just do not get the science. . . . The impulse to dismiss public views as simply ill-informed is not only itself ill-informed but is problematic because it deprives society of the freedom to decide what forms of progress are culturally and morally acceptable.”^48^

## A Shortcoming of the Democratic Approach

Unfortunately, the democratic solutions advocated by Baylis and Jasanoff et al. also have a serious shortcoming. If one of the problems with the elitist approach was that it sacrificed democratic values for the sake of imposed rationally inferred moral values, the problem with the democratic model is rather the opposite one: it sacrifices reasoned and well-informed decision-making for the sake of democratic values. The problem with Baylis’ proposal is that due to the fundamental moral disagreement at the level of substantive ethical questions, it is likely that a “broad based participation by persons from around the world with a range of perspectives and interests” will lead to fragmentation rather than to the widespread consensus that Baylis advocates. In addition to this, while it is true that Baylis does take into account certain conditions that need to be met in order to achieve her particular conception of consensus,^49^ consensus may not be the most desirable aim to pursue, both because it may be a “mask hiding relations of domination and exclusion”^50^ and because it might be reached “to the detriment of opponents or the recalcitrant who have been unable to express themselves or who have been silenced.”^51^ The problem with the proposal of Jasanoff et al.—we contend—is instead that it challenges the very idea of expertise and with it, the idea of relying on experts. This is problematic as many people’s decisions may be uninformed or, if we may believe Greene, based on automatically formed and intuitive responses.

## The Enlightened Democracy Approach

We propose that regulations for genome editing ought to be developed on the basis of what we call an “enlightened democracy” approach, which, in our view, includes the strengths of the elitist approach and the democratic approach suggested by Baylis and Jasanoff et al., while avoiding, to the greatest extent possible, their shortcomings. The enlightened democracy approach to regulating genome editing relies partly on Greene’s ideas of a shared moral standard and the relevance of experts in policymaking, and partly on the literature on deliberative democracy.^52^ At the same time, our proposed approach takes up the challenges raised by Baylis, and especially by Jasanoff et al., in favor of democratic deliberation and broad-based public engagement. Our proposal is enlightened, in that it aims to include the various views of different categories of experts, and democratic, in that it aims to open up the debate to various sorts of nonexperts and engage with public views on genome editing.

The first characteristic of our approach is that it rejects an agenda for genome editing that is solely based on what experts define as worth pursuing. At the same time, it grants experts an ancillary but necessary role in the development of such an agenda. Building on the work of Philip Kitcher, we argue that the policies regulating genome editing research and implementation should strive towards the ideal of “well-ordered science.” According to Kitcher, scientific research and clinical applications are well-ordered when their agendas coincide with ideal deliberators’ judgments and world views, which in turn are representative of the diversity of judgments and world views coexisting in a given community. In the context of genome editing and its applications, this ideal entails that such applications are well-ordered only if they align with what people—coming together and discussing their values and preferences—would decide in a deliberative process. The deliberations among people aim to provide “the most justifiable conception for dealing with moral disagreement in politics.”^53^ This means that, as we saw above, even if disagreement often cannot be avoided, people’s preferences should be taken into account in order to avoid distrust and illegitimacy. In addition, deliberations among peers facilitated and informed by experts allow that preferences are perfected and epistemic flaws ironed out. A deliberative process that involves ordinary people as well as experts seems to us the most desirable strategy on two desiderata, namely “the degree to which policy outcomes match the substantive goals of society in question; and the degree to which they achieve normatively justifiable ends.”^54^

The second characteristic of our proposed approach is that it sets certain background conditions to participating in these deliberations.^55^ Contrary to the proposals such as those of Jasanoff et al., and also James Ben Hurlbut, people entering these deliberations should meet certain criteria in order to avoid the two dangers outlined above (i.e., regulations that do not match societal goals and that do not achieve normatively justifiable ends). Deliberators need to meet “epistemic conditions”^56^ of mutual engagement, which require deliberators to not rely on false beliefs about the world, to be aware of the consequences of the debated matter for one another, and to know preferences and wishes of other deliberators. With respect to genome editing, this means that deliberators should gain a basic knowledge of the functioning, potential uses, potential risks, and potential benefits of genome editing. Scientific experts, as well as social and technology studies experts, sociologists, philosophers, and lawyers would assist in bringing to light expected consequences of permissive or restrictive regulations for genome editing and make sure that deliberators can fulfil such epistemic conditions.

Other conditions for deliberators to take part in these discussions are “affective”^57^ in that deliberators will be required to work towards the “expansion of one’s sympathies, in which the perceived desires of those with whom one deliberates are given equal weight with one’s own.”^58^ These affective conditions of mutual engagement reflect also deliberative democracy’s background conditions of mutual respect.^59^ Only if both conditions apply is the process one of genuinely mutual engagement.

Moreover, epistemic and affective conditions allow for the emergence and especially the discussion of “tutored” as opposed to “raw” preferences.^60^ There is significant disagreement among experts about substantive ethical questions regarding genome editing. These differences are likely to be equally found in wider society, where a plurality of values obtain.^61^ Hence, deliberators may have different preferences with respect to regulations, and their judgments may be influenced by these preferences when they come together and discuss different possible routes for scientific research and applications. The preferences that these individuals discuss should, however, not be “raw” preferences influenced by whatever inclination or temporary impulse these individuals are subjected to; in other words, the preferences should not be devoid of any background information, but rather “tutored preferences”: preferences informed by the current state of the art of the matter and especially by the significance that potential applications of the technology in question may have for people’s lives.^62^ In addition to this, these preferences should be tutored in the sense that they will be perfected in a discussion with experts and in a discussion with epistemic peers (e.g., other members of the public participating in the deliberative processes).

In our view, these characteristics enable a deliberative process to take place, one that avoids what in our view are the most problematic shortcomings of the elitist and democratic approaches to regulating genome editing. The enlightened democracy approach could be criticized on practical and ethical grounds too, but we contend that its shortcomings should be factored against the benefits and the shortcomings of the alternatives thus far proposed. From a practical point of view, our proposed approach may still generate or fail to solve disagreement. Disagreement at the first level (the substantive-ethical) and disagreement at the second level (the ethical-political) are interlinked and mutually influenced. However, even if there is lingering disagreement, our approach will reduce the risk of stifling policy-making processes, as at least epistemic flaws will have been mitigated and the different moral beliefs and preferences discussed. As argued by Philip Kitcher,^63^ Amy Gutmann and Dennis Thompson,^64^ Joshua Cohen,^65^ and—from a different perspective— Jonathan Haidt,^66^ the give-and-take of preferences and judgments allows for addressing misapprehensions and for appreciating the value of moral beliefs different from our own. In the best-case scenario, recognizing the value of other points of view will help deliberators to engage with these points of view and perhaps to reflect on their own moral beliefs. This could help the activation of Greene’s manual mode and allow for a reasoned reflection to emerge. In some cases, the disagreement will not be resolved and the debate will remain polarized, but the mutual engagement would hopefully mitigate legitimacy problems and distrust. Our proposed approach will be criticized by those who would grant more “power to the people” and those who are wary of any involvement of experts as they predetermine the questions that are worth pursuing and hence limit the scope and type of questions that are discussed in these deliberations.^67^ It will be also criticized by those who are wary of involving the public in discussions concerning new technologies and how they should be regulated. Without entering in a complicated discussion with both sides on burden of proof, we contend that our approach accommodates these competing views better than the alternatives.

## Conclusion

In this paper, we have proposed an approach to addressing ethical-political questions regarding genome editing—i.e., questions about how genome editing should be regulated in the face of deep and persistent disagreement about substantive ethical questions. We have sketched a possible elitist approach grounded in the metamorality proposal of Greene and based on the deliberation of some sort of experts, and then discussed the democratic approach proposed by Baylis and Jasanoff et al. We have argued that the approaches each have strengths but also significant shortcomings. We have then proposed a new approach—the “enlightened democracy” approach—that aims to reconcile the need for a democratic engagement involving mutual respect for competing views on the one hand and a well-informed discussion on the other. Our proposal is meant to sketch a theoretical framework to inform the ethical debate on how to regulate genome editing. We appreciate that our proposed approach would need to be further developed and refined. Most notably, we have not addressed the question of how such an approach would translate into practice. In this sense, our paper is situated within the scholarship in moral and political philosophy that proposes approaches to regulate new technologies in pluralistic and democratic societies. We believe, however, that a study of the implementation of the enlightened democracy approach would be worth pursuing, perhaps in another paper.

## Notes

1. GutmannA, ThompsonDF. Democracy and Disagreement. Cambridge, MA: The Belknap Press of Harvard University Press; 1996.

2. MillJS. On Liberty. London: Dent; 1979 (Original work published in 1859).

3. House of Lords, Selected Committee on Science and Technology. *Science and Society*. London: Her Majesty’s Stationary Office; 2000.

4. Adam B, Berkhout F, Dyson T, Grove-White R, Marsden T, O’Riordan T, et al. *The Politics of GM Food: Risk, Science and Public Trust*. University of Sussex: ESRC Global Environmental Change Programme, Special Briefing; 1999; Wynne BE. The Times Higher Education Supplement. *Patronising Joe Public*; 1996 Apr 12; available at https://www.timeshighereducation.com/news/patronising-joe-public/93081.article (last accessed 6 March 2018).

5. Ibid. See also note 3, House of Lords 2000.

6. Jasanoff S, Hurlbut JB, Saha K. CRISPR democracy: Gene editing and the need for inclusive deliberation. *Issues in Science and Technology* 2015;32(1):37–49.

7. Ledford H. CRISPR, the disruptor. *Nature* 2015;522:20–4.

8. See, e.g., CavaliereG. Genome editing and assisted reproduction: Curing embryos, society or prospective parents? Medicine, Health Care and Philosophy 2018;21(2):215–25;10.1007/s11019-017-9793-yPMC595605228725950 GyngellC, DouglasT, SavulescuJ. The ethics of germline gene editing. Journal of Applied Philosophy 2017;34(4):498–513.2891965510.1111/japp.12249PMC5573992

9. BaylisF. Human germline, genome editing and broad societal consensus. Nature Human Behaviour 2017;1:1–3. LanphierE, UrnovF, HaeckerSE, WernerM, SmolenskiJ. Don’t edit the human germ line. Nature News 2015;519(7544):410–1.10.1038/519410a25810189

10. KassLR. Life, Liberty and the Defense of Dignity: The Challenge for Bioethics. San Francisco: Encounter books; 2004; SandelM. The case against perfection. The Atlantic Monthly 2004;293(3):51–62.15468473

11. For an analysis of ‘playing God’ arguments see, for instance, Savulescu J, Brostrom N, Coady CAJ. Playing God. In: Savulescu J, Bostrom N, eds*. Human Enhancement*. Oxford, UK: Oxford University Press; 2009:155–80.

12. Some have argued that gene editing could lead back to eugenics: King D. Editing the human genome brings us one step closer to consumer eugenics. *The Guardian* 2017 Aug 4; available at https://www.theguardian.com/commentisfree/2017/aug/04/editing-human-genome-consumer-eugenics-designer-babies (last accessed 5 Mar 2018); Pollack R. Eugenics lurk in the shadow of CRISPR. *Science* 2015;348(6237):871.

13. With some exceptions: see note 9, Baylis 2017; see note 6; Jasanoff S, Hurlbut JB, Saha K. 2015; Baylis F. ‘Broad societal consensus’ on human germline editing. *Harvard Health Policy Review* 2016;15(2):19–22; Hurlbut JB. Limits of responsibility: Genome editing, Asilomar, and the politics of deliberation. *Hastings Center Report* 2015;45(5):11–4; Chan S, Arellano MM. Genome editing and international regulatory challenges: Lessons from Mexico. *Ethics, Medicine and Public Health* 2016;2(3):426–34.

14. See note 6, Jasanoff et al. 2015.

15. See note 9, Baylis 2017.

16. See, for instance, note 1, Gutmann, Thompson 1996; Cohen J. Deliberation and democratic legitimacy. In: Hamlin A, Petitt P, eds. *The Good Polity*. Oxford, UK: Blackwell; 1989:17–34; Gutmann A, Thompson D. *Why Deliberative Democracy?* Princeton, NJ: Princeton University Press; 2009; Kitcher P. *Science, Truth and Democracy*. New York: Oxford University Press; 2003; Kitcher P. *Science in a Democratic Society*. Amherst, NY: Prometheus Books; 2011.

17. See, e.g., Harris J. Why human gene editing must not be stopped. *The Guardian* 2015 Dec 2; available at https://www.theguardian.com/science/2015/dec/02/why-human-gene-editing-must-not-be-stopped (last accessed 6 Mar 2018) and Savulescu J, Pugh J, Douglas T, Gyngell C. The moral imperative to continue gene editing research on human embryos. *Protein & Cell* 2015;6(7):476–9.

18. See, e.g., note 9, Lanphier E et al. 2015; Collins FS. *Statement on the NIH funding of research using gene-editing technologies in human embryos*; available at http://www.nih.gov/about-nih/who-we-are/nih-director/statements/statement-nih-funding-research-using-gene-editing-technologies-human-embryos (last accessed 6 Mar 2018); and UNESCO International Bioethics Committee*. UNESCO panel of experts calls for ban on “editing” of human DNA to avoid unethical tampering with hereditary traits*; available at http://en.unesco.org/news/unesco-panel-experts-calls-ban-editing-human-dna-avoid-unethical-tampering-hereditary-traits (last accessed 6 March 2018).

19. Often, moral disagreement persists even in ideal conditions, among people that are well-informed and fully rational. This is what Doris and Plakias (2008) refer to as “fundamental disagreement,” which obtains even when ideal conditions are met. See Doris JM, Plakias A. How to argue about disagreement: Evaluative diversity and moral realism. In: Sinnott-Armstrong W, ed. *Moral Psychology, Volume 2: The Cognitive Science of Morality: Intuition and Diversity*. Cambridge, MA: The MIT Press; 2008:303–31.

20. Spinoza, *Ethics*, 1677 book IV, prop. XVII.

21. Rawls J. *Political Liberalism*. New York: Columbia University Press; 1993.

22. GreeneJ. Moral Tribes. London: Atlantic Books; 2014; HaidtJ. The Righteous Mind: Why Good People are Divided by Politics and Religion. New York: Pantheon; 2012. See also: Haidt J, Bjorklund F. Social intuitionists answer six questions about moral psychology. In: Sinnott-Armstrong W, ed. *Moral Psychology, Volume 2: The Cognitive Science of Morality: Intuition and Diversity*, Cambridge, MA: The MIT Press; 2008:181–217.

23. See, for instance, Kahane G, Shackel N. Methodological issues in the neuroscience of moral judgement. *Mind & Language* 2010;25(5):561–582; Nagel T. You can’t learn about morality from brain scans: The problem with moral psychology. *The New Republic* 2013 Nov 2; available at https://newrepublic.com/article/115279/joshua-greenes-moral-tribes-reviewed-thomas-nagel (last accessed 6 March 2018); Wright R. Why can’t we all just get along? The uncertain biological basis of morality. *The Atlantic* 2013 Nov; available at https://www.theatlantic.com/magazine/archive/2013/11/why-we-fightand-can-we-stop/309525/ (last accessed 6 Mar 2018).

24. Foot P. The problem of abortion and the doctrine of double effect. *Oxford Review* 1967;5:5–15; Thomson JJ. Killing, letting die, and the trolley problem. *The Monist* 1976;59(2):204-17; Thomson JJ. The trolley problem*. The Yale Law Journal* 1985;1;94(6):1395–415.

25. This is similar to the dual-process model that other psychologists developed with respect to the way humans make judgments more generally, see, for instance, Kahneman D. *Thinking, Fast and Slow*. New York: Farrar, Straus and Giroux; 2011.

26. See also note 22, Haidt 2012.

27. The automatic mode is activated in the trolley problem scenario in which we can save five people at the cost of killing one without having to actively push someone onto the tracks, something that many people intuitively reject.

28. See note 22, Greene 2014, at 290.

29. While the very same notion of “rationality” is itself difficult to define in philosophical terms, in psychological terms we can define practical rationality as the capacity to make decisions that are not based merely on unanalyzed intuitions and emotions. In Greene’s words, “Reasoning, as applied to decision making, involves the conscious application of decision rules. . . . Reasoning frees us from the tyranny of our immediate impulses by allowing us to serve values that are not automatically activated by what’s in front of us,” see note 22 Greene 2014 at 13.

30. See note 22, Greene 2014, at 15.

31. Ibid.

32. There is a helpful Wright (2013) analogy of metamorality as “the moral equivalent of Esperanto,” see note 23, Wright R 2013.

33. See note 22, Greene 2014, at 201.

34. This view that is shared, for instance, by Singer P. *The Expanding Circle*. New York: Farrar, Straus and Giroux; 1981 and Sidgwick H. *The Methods of Ethics, 7th ed*. London: Macmillan; 1907.

35. There is a literature, both in philosophy and within bioethics specifically, about what moral expertise is, whether it exists at all, and who counts as a moral expert, with which we do not have the space to engage here. For the purposes of our paper, however, what matters is not so much how moral expertise should be defined and whether it exists, but the fact that the elitist model we discuss would have to rely on some sort of moral expertise—as well as on other kinds of expertise—rather than on the active participation of the population or of some representation of it.

36. As Peter Singer says in his defense of moral expertise, “someone familiar with moral concepts and with moral arguments, who has ample time to gather information and think about it, may reasonably be expected to reach a soundly based conclusion more often than someone who is unfamiliar with moral concepts and moral arguments and has little time.” See Singer P. Moral experts. *Analysis* 1972;32(4):115–117, at 117. Similarly, Harris and Lawrence (2018) argued that “‘ Ethical expertise’ if and where it exists must consist principally in the ability to create, assemble, analyze or present the combination of evidence and argument required to establish, defend, qualify, weaken or demolish a proposition of ethical significance. Or, to reveal problems or ambiguities, contradictions or inconsistencies, in short strengths and weaknesses, in ethical positions, judgments, claims or conclusions”; Harris J, Lawrence D. Ethical expertise and public policy. In: Lever A, Poama A, eds. *Routledge Handbook of Ethics and Public Policy*. Abingdon, UK: Routledge; forthcoming.

37. For instance, these arguments would apply to elitist approaches based on the alleged moral expertise of religious authorities.

38. See note 3, UK House of Lords 2000.

39. Archard D. Why moral philosophers are not and should not be moral experts. *Bioethics* 2011;25(3):119–27, at 20.

40. Here we follow Dryzek’s (2001) definition of legitimacy, namely that “outcomes are legitimate to the extent that they receive reflective assent through participation in authentic deliberation by all those subject to the decision in question.” Dryzek J. Legitimacy and economy in deliberative democracy. *Political Theory* 2001;29(5):651–669, at 651.

41. O’Neill O. *A Question of Trust: The BBC Reith Lectures 2002*. Cambridge, UK: Cambridge University Press; 2002; Resnik D. Scientific research and the public trust. *Science and Engineering Ethics* 2011;17(3):399–409.

42. See note 9, GutmannThompson 2009; note 13, Chan, Arellano 2016; and Parkinson J. Legitimacy problems in deliberative democracy. Political Studies 2003;51(1):180–96.

43. This view was already defended by John Stuart Mill in his defense of freedom of speech of people with different moral views, see note 2 Mill 1979/1859.

44. See note 6, JasanoffHurlbut, Saha 2015; see note 9, Baylis 2017; see note 13, Baylis 2016; and Jasanoff F, Hurbut JB. A global observatory for gene editing. Nature 2018;555(7697):435–7.3203437310.1038/d41586-018-03270-w

45. The Royal Society—a U.K. learned society—commissioned both a public dialogue deliberative process (2013) and launched a survey (2017) on genetic technologies’ applications to plants, humans (heritable and nonheritable), nonhuman animals (pets, wild animals, and animals used as source of food). A report of the survey and of the public dialogue is available at https://royalsociety.org/~/media/policy/projects/gene-tech/genetic-technologies-public-dialogue-hvm-full-report.pdf (last accessed 25 Apr 2018).

46. The National Academies of Sciences Engineering Medicine. *On Human Gene Editing: International Summit Statement*; Available at http://www8.nationalacademies.org/onpinews/newsitem.aspx?RecordID=12032015a (last accessed 6 Mar 2018).

47. See note 13, Baylis 2016, at 22.

48. See note 6, Jasanoff S, Hurlbut JB, Saha K 2015; elsewhere—and in a similar fashion—Jasanoff and Hurlbut have called for a “global observatory on gene editing” and argued that “free enquiry, the lifeblood of science, does not mean untrammeled freedom to do anything. Society’s unwritten contract with science guarantees scientific autonomy in exchange for a research enterprise that is in the service of, and calibrated to, society’s diverse conceptions of the good. As the dark histories of eugenics and abusive research on human subjects remind us, it is at our peril that we leave the human future to be adjudicated in biotechnology’s own ‘ecclesiastical courts.’” See note 44, Jasanoff, Hurbut 2018, at 437.

49. Baylis (2016) does not really define what she means by consensus, but she is adamant in stressing that, “consensus does not mean that everyone thinks that the decision made is necessarily the best one possible. . . . What it does mean is that in coming to that decision on one felt that her position on the matter was misunderstood or that it wasn’t given a proper hearing”; See note 6, Baylis 2016, at 22. The author also lays the condition of mutual engagement for achieve such consensus (responsibility, self-discipline, respect, cooperation, struggle).

50. CallonM, LascoumesP, BartheY. Acting in an Uncertain World. Cambridge, MA: MIT Press; 2001, at 4.

51. Ibid. quoting S. Jasanoff.

52. In particular, Gutmann and Thompson (1996, 2009), see note 9; Gutmann, Thompson 1996, 2009; and see note 16, Kitcher 2003, at 2011.

53. See note 16, Gutmann, Thompson 2009, at 10.

54. See note 42, Parkinson 2003, at 183.

55. See note 16, Gutmann, Thompson 2009; and Kitcher 2011.

56. See note 16, Kitcher 2011

57. Ibid.

58. Ibid., 51.

59. See note 16, Gutmann, Thompson 2009.

60. See note 16, Kitcher 2003, at 2011.

61. See note 22, Haidt 2012; NagelT. Mortal Questions. Cambridge, UK: Cambridge University Press; 1979 (in particular chapter 9, The Fragmentation of Value).

62. See note 16, Kitcher 2003, at 118.

63. See note 16, Kitcher 2003, at 2011.

64. See note 1, Gutmann, Thompson 1996; see note 16, Gutmann, Thompson 2009.

65. See note 16, Cohen 1989.

66. See note 16, Kitcher 2011.

67. HurlbutJB. Experiments in Democracy: Human Embryo research and the Politics of Bioethics. New York: Columbia University Press; 2017.

